# The authors reply: Comment on: "Aldehyde dehydrogenases contribute to skeletal muscle homeostasis in healthy, aging, and Duchenne muscular dystrophy patients" by Etienne et al.

**DOI:** 10.1002/jcsm.12629

**Published:** 2020-09-17

**Authors:** Jessy Etienne, Pierre Joanne, Cyril Catelain, Stéphanie Riveron, Alexandra Bayer Wildberger, Jérémy Lafable, Isabel Punzon, Stéphane Blot, Onnik Agbulut, Jean‐Thomas Vilquin

**Affiliations:** ^1^ Department of Bioengineering and QB3 Institute University of California, Berkeley Berkeley CA USA; ^2^ Sorbonne Université, INSERM, AIM, Centre de Recherche en Myologie, UMRS 974, AP‐HP Paris France; ^3^ Sorbonne Université, CNRS, INSERM, Institut de Biologie Paris‐Seine, IBPS, UMR 8256 Paris France; ^4^ Université Paris‐Est Créteil, INSERM, Institut Mondor de Recherche Biomédicale, IMRB, Ecole Nationale Vétérinaire d'Alfort, ENVA, U955‐E10 Maisons‐Alfort France; ^5^ Sorbonne Université, INSERM, AIM, Centre de Recherche en Myologie, UMRS 974, CNRS, AP‐HP Paris France

We wish to thank Drs Campos, Chen, and Ferreira for their careful and subtle appraisal of this study, for their rewarding proposals and discussion, and for underlining the perspectives. Our recent article^1^ was the first step in investigating the complex contribution of aldehyde dehydrogenases (ALDHs) in skeletal muscle's homeostasis, regeneration, and myopathies.

As presented by these authors, Aldefluor^®^ assay (ALDEF) alone cannot be considered a univocal marker for the analysis of heterogeneous populations of cells, and this is not specific to the skeletal muscle tissue. Indeed, the measurement of ALDEF relies on enzymatic activities, and there are still uncertainties regarding the metabolism of ALDEF by several or most ALDH isoenzymes, due to their structural evolution and diversity. All isoenzymes are not equivalent. Based on gene transfer experiments, nine isoenzymes have been recently proposed to metabolize ALDEF efficiently (ALDH1A1, ALDH1A2, ALDH1A3, ALDH1B1, ALDH2, ALDH3A1, ALDH3A2, ALDH3B1, and ALDH5A1),^2^ and the article^1^ suggested that most of them were involved in muscle biology. New probes with dedicated isoenzyme selectivity (thiophene‐bridged aldehydes^3^), or complementary to ALDEF (Aldered^4^), have been described or are under development. ALDEF labelling may be necessary but not sufficient to segregate subpopulations of cells. We then combined ALDEF labelling with known extracellular markers to improve the definition of cell types. Such combinations have been used in different fields such as haematology.^5^ Previously, we distinguished two human muscle cell populations with ALDH activity (ALDEF^+^/CD34^+^ and ALDEF^+^/CD34^−^) but a different intrinsic ability to engage in a myogenic programme.^6^ In the study,^1^ we investigated how these two main subpopulations are influenced by ageing and Duchenne muscular dystrophy (DMD), and we pinpointed important differences between healthy donors and DMD patients.

Relating to DEAB, it is a reference inhibitor provided with the Aldefluor^®^ kit assay. It presents different selectivities, turnover, and mechanisms regarding the inhibition of the several ALDH isoenzymes, and it cannot cover their whole spectrum. This may be taken in consideration for fine tuning of mechanistic experiments *in vitro* and *in vivo*.^2^, ^7^, ^8^ In the article,^1^ DEAB was used classically as a negative control for ALDEF labelling. Other classes of inhibitors exist to perform functional assays with different selectivity and enzymatic features (reversible, irreversible, competitive, and non‐competitive).^7^, ^8^ Extensive researches of modulators are done in the field of cancerology, since ALDHs are involved in the resistance to some chemotherapy agents^9^, ^10^, ^11^, ^12^ and in the field of cardiology where ALDH2 activation by dedicated molecules improves post‐infarction remodelling as underlined by Drs Campos, Chen, and Ferreira.^13^, ^14^ The identification of specific modulators is and will be extremely valuable to cross‐fertilize the research in the field of muscle physiology, regeneration, and fibrosis.

ALDEF may be very useful to prepare cells on important scales for the purpose of regenerative medicine but may present limitations regarding fundamental biology. This prompted us to look directly at the expression of isoenzymes, using histochemistry and molecular biology. The analysis of tissue distribution of ALDH isoenzymes emphasized the presence of several cell types linked to different functions afferent to muscle physiology: myogenesis, angiogenesis, and neural and stromal support. It revealed also differential presence of ALDH3A2, ALDH1L1, and ALDH9A1 between tissues from healthy and DMD patients, and some ALDH isoenzymes may become useful markers. Finally, the selective expression of some isoenzymes in crude tissue, dissociated mononucleated cells, and myogenic cultures suggests their involvement at several levels of muscle homeostasis. Interestingly, most of them, but not all, are able to metabolize ALDEF.^2^

The article^1^ is primarily descriptive and does not provide direct mechanistic roles for ALDH isoenzymes in skeletal muscle regeneration and physiopathology. Indeed, ALDH isoenzymes are expressed in different cellular compartments and involved in several biochemical pathways whose link with muscle physiology is not always established. While the production of retinoic acid by some isoenzymes is involved in skeletal and cardiac muscle development, and the detoxification of several aldehydes' adducts plays roles in muscle homeostasis in the context of muscular dystrophies, ageing, or sarcopenia, the potential roles played by several ALDH metabolites (folate, glutamate, and succinate) have still to be explored in muscle. Nevertheless, the article^1^ documented the involvement of ALDH1A3, ALDH1B1, ALDH2, ALDH3A2, ALDH7A1, ALDH8A1, and ALDH9A1, at least, in addition to ALDH1A1 whose expression in muscle tissue has been previously documented.

Altogether, the use of targeted activators or inhibitors (pharmacological agents discussed above, siRNA, CRISPR‐Cas control of gene expression, etc.) in step‐by‐step experiments *in vitro* and *in vivo* will help in understanding the respective contribution and complementarity of the isoenzymes expressed in skeletal muscle, to promote a better knowledge of muscle physiology and a better control of muscle pathology. We thank Drs Campos, Chen, and Ferreira who have shed light on the importance of ALDH in muscle tissue in these perspectives.

The authors of this manuscript certify that they comply with the ethical guidelines for authorship and publishing in the *Journal of Cachexia, Sarcopenia and Muscle*.^15^

## References

[1] Etienne J , Joanne P , Catelain C , Riveron S , Bayer AC , Lafable J , et al. Aldehyde dehydrogenases contribute to skeletal muscle homeostasis in healthy, aging, and Duchenne muscular dystrophy patients. J Cachexia Sarcopenia Muscle 2020;11:1047–1069. 32157826 10.1002/jcsm.12557 PMC7432589

[2] Zhou L , Sheng D , Wang D , Ma W , Deng Q , Deng L , et al. Identification of cancer‐type specific expression patterns for active aldehyde dehydrogenase (ALDH) isoforms in ALDEFLUOR assay. Cell Biol Toxicol 2019;35:161–177. 30220009 10.1007/s10565-018-9444-y PMC6424948

[3] Maity S , Sadlowski CM , George Lin JM , Chen CH , Peng LH , Lee ES , et al. Thiophene bridged aldehydes (TBAs) image ALDH activity in cells *via* modulation of intramolecular charge transfer. Chem Sci 2017;8:7143–7151. 29081945 10.1039/c7sc03017g PMC5635522

[4] Dollé L , Boulter L , Leclercq IA , van Grunsven LA . Next generation of ALDH substrates and their potential to study maturational lineage biology in stem and progenitor cells. Am J Physiol Gastrointest Liver Physiol 2015;308:G573–G578. 25656041 10.1152/ajpgi.00420.2014 PMC4385895

[5] Storms RW , Green PD , Safford KM , Niedzwiecki D , Cogle CR , Colvin OM , et al. Distinct hematopoietic progenitor compartments are delineated by the expression of aldehyde dehydrogenase and CD34. Blood 2005;106:95–102. 15790790 10.1182/blood-2004-09-3652 PMC1895136

[6] Vauchez K , Marolleau JP , Schmid M , Khattar P , Chapel A , Catelain C , et al. Aldehyde dehydrogenase activity identifies a population of human skeletal muscle cells with high myogenic capacities. Mol Ther 2009;17:1948–1958. 19738599 10.1038/mt.2009.204 PMC2835039

[7] Koppaka V , Thompson DC , Chen Y , Ellermann M , Nicolaou KC , Juvonen RO , et al. Aldehyde dehydrogenase inhibitors: a comprehensive review of the pharmacology, mechanism of action, substrate specificity, and clinical application. Pharmacol Rev 2012;64:520–539. 22544865 10.1124/pr.111.005538 PMC3400832

[8] Morgan CA , Parajuli B , Buchman CD , Dria K , Hurley TD . *N*,*N*‐Diethylaminobenzaldehyde (DEAB) as a substrate and mechanism‐based inhibitor for human ALDH isoenzymes. Chem Biol Interact 2015;234:18–28. 25512087 10.1016/j.cbi.2014.12.008 PMC4414715

[9] Belmont‐Díaz JA , Calleja‐Castañeda LF , Yoval‐Sánchez B , Rodríguez‐Zavala JS . Tamoxifen, an anticancer drug, is an activator of human aldehyde dehydrogenase 1A1. Proteins 2015;83:105–116. 25354921 10.1002/prot.24709

[10] Chefetz I , Grimley E , Yang K , Hong L , Vinogradova EV , Suciu R , et al. A pan‐ALDH1A inhibitor induces necroptosis in ovarian cancer stem‐like cells. Cell Rep 2019;26:3061–3075.e6. 30865894 10.1016/j.celrep.2019.02.032 PMC7061440

[11] Pors K , Moreb JS . Aldehyde dehydrogenases in cancer: an opportunity for biomarker and drug development? Drug Discov Today 2014;19:1953–1963. 25256776 10.1016/j.drudis.2014.09.009

[12] Yang SM , Martinez NJ , Yasgar A , Danchik C , Johansson C , Wang Y , et al. Discovery of orally bioavailable, quinoline‐based aldehyde dehydrogenase 1A1 (ALDH1A1) inhibitors with potent cellular activity. J Med Chem 2018;61:4883–4903. 29767973 10.1021/acs.jmedchem.8b00270 PMC6004562

[13] Chen CH , Ferreira JC , Gross ER , Mochly‐Rosen D . Targeting aldehyde dehydrogenase 2: new therapeutic opportunities. Physiol Rev 2014;94:1–34. 24382882 10.1152/physrev.00017.2013 PMC3929114

[14] Gomes KM , Campos JC , Bechara LR , Queliconi B , Lima VM , Disatnik MH , et al. Aldehyde dehydrogenase 2 activation in heart failure restores mitochondrial function and improves ventricular function and remodelling. Cardiovasc Res 2014;103:498–508. 24817685 10.1093/cvr/cvu125 PMC4155470

[15] von Haehling S , Morley JE , Coats AJS , Anker SD . Ethical guidelines for publishing in the *Journal of Cachexia, Sarcopenia and Muscle*: update 2019. J Cachexia Sarcopenia Muscle 2019;10:1143–1145. 31661195 10.1002/jcsm.12501 PMC6818444

